# A Novel Lymphosome-Based Long-Lasting Rat Tail Model of Lymphedema

**DOI:** 10.1055/a-2687-0506

**Published:** 2025-09-03

**Authors:** Junzhe Chen, Yun Wang, Shilin Tu, Yan Zhou, Haokun Qin, Zeyao Tang, Yuezhong Chen, Shune Xiao, Chengliang Deng

**Affiliations:** 1 Department of Burns and Plastic Surgery, Affiliated Hospital of Zunyi Medical University, Zunyi, Guizhou, China; 2 The Collaborative Innovation Center of Tissue Damage Repair and Regeneration Medicine, Zunyi Medical University, Zunyi, Guizhou, China

**Keywords:** lymphedema, animal model, tail

## Abstract

**Background:**

Secondary extremity lymphedema is a chronic and progressive condition caused by obstructed lymphatic drainage, commonly following lymphadenectomy, infection, or trauma. Rodent models are preferred for experimental lymphedema research due to cost-effectiveness and reproducibility. Currently, rat tail models encounter limitations due to transient swelling and their inability to fully replicate the comprehensive pathophysiology of lymphedema, particularly concerning the effects of lymph node removal.

**Methods:**

We developed a series of rat tail lymphedema models incorporating skin resection, deep lymphatic vessel disruption, and gluteal lymph node (GLN) excision to assess effects on lymphatic architecture, inflammation, and fibrosis. Indocyanine green (ICG) lymphography was used to visualize lymphatic function. Tail volume and circumference were measured weekly, and histological assessments quantified fibrosis and fibroadipose thickening. Bulk RNA sequencing was performed to characterize the inflammatory processes triggered by GLN removal.

**Results:**

The combined model (skin removal, deep lymphatic disruption, and GLN excision) resulted in severe and persistent lymphedema marked by progressive swelling and pronounced fibrosis. ICG lymphography confirmed disruption of superficial lymphatic flow with partial recanalization of deep channels. Notably, lymphangiogenesis was observed at the GLN excision site, forming compensatory pathways connecting the tail to the popliteal lymph nodes. Histology revealed extensive collagen deposition and fibroadipose thickening in groups with lymph node removal, with the combined model showing the most pronounced changes. Bulk RNA-sequencing confirmed the removal of GLN involving the inflammatory and fibrosis process in the tail model.

**Conclusion:**

This lymphosome-based rat tail model successfully replicates key features of lymphedema, including sustained swelling, lymphatic disruption, inflammation, and fibrosis.

Secondary extremity lymphedema is a chronic, progressive condition resulting from obstructed lymphatic drainage due to factors such as infection, trauma, or malignancy. This obstruction causes lymphatic fluid to accumulate in the interstitial spaces, triggering a cascade of pathophysiological changes. 1

Animal models that accurately replicate lymphedema pathophysiology are indispensable for collecting data and testing hypotheses related to disease mechanisms and therapeutic strategies. Although various models have been developed using rabbits, sheep, dogs, and pigs, rodents remain the preferred option due to their cost-effectiveness and reproducibility. 2 3 Rat is widely used to develop animal models for simulating human conditions. 4 However, current rat tail models are limited: The induced lymphedema is typically transient and fails to capture the full pathophysiological features seen in human disease. 5 These models usually involve blocking both deep and superficial lymphatic drainage but ignoring lymph node manipulation, despite the clinical observation that lymph node dissection is a critical factor in lymphedema onset. 6 7

Anatomical studies of the rat lymphatic system highlight the pivotal role of regional lymph nodes in drainage and their delineation of distinct lymphatic territories, or lymphosomes. 8 In particular, the gluteal lymph nodes (GLNs) are crucial for tail lymphatic drainage, making them central to the rat tail lymphedema model. Incorporating lymph node involvement is significant not only because these nodes regulate fluid flow but also because they shape the immune response to lymphatic dysfunction. 9 The gluteal lymphosome's consistent anatomical organization and its linkage to the tail's lymphatic network emphasize its value in mimicking clinical lymphedema—especially in contexts where lymph node dissection exacerbates the disease.

A rat tail model that produces persistent lymphedema and more accurately mirrors its pathophysiological changes would therefore greatly advance research into lymphedema mechanisms and interventions. In this study, we sought to establish a lymphosome-based reproducible, long-lasting rat model of lymphedema that closely aligns with the clinical manifestations of human disease.

## Methods

### Animals

Female Sprague–Dawley rats (350–400 g) were provided by Enswell Biotechnology Co. (Chongqing, China). All animal procedures were conducted in accordance with protocols approved by the Animal Care and Use Committee of Zunyi Medical University. Rats were divided into different groups ( *n* = 6); the LN group: Rats with removal of GLN only; the Sup group: Rats with removal of tail skin only; the Sup + Deep group: Rats with removal of tail skin and ligation of deep lymphatic vessels (LVs); the Sup + LN group: Rats with removal of both GLN and tail skin; the Sup + Sham group: Rats with the removal of the tail skin, while finding and preserving GLN; the Sup + Deep + LN group: Rats with removal of both GLN and tail skin combined with ligation of deep LVs; and the Sup + Deep + Sham group: Rats with removal of tail skin and ligation of deep LVs, while finding and preserving GLN. It is worth noting that no mortality or tail ischemia occurred in any of the groups, and no animals were excluded due to necrosis or other complications. In all groups, rats were anesthetized with 2.5% isoflurane during the procedure.

### Procedure for Identification and Ligation of Tail Lymphatic Vessels

**Video 1** Evans' blue dye to visualize the lymphatic vessels.


The superficial lymphatic channels of the tail were disrupted by creating a circumferential skin incision and excising an 8-mm wide strip of skin, located 2 cm distal to the base of the tail ( Supplementary Figs. S1 and S2 [available in the online version only]). The 8-mm excision width was selected based on literature reports where most rat tail models use widths >5 mm, and some up to 10 mm. As no standard exists, we chose 8 mm as a practical midpoint to balance lymphatic disruption and tissue preservation. 10 11 The deep LVs, positioned adjacent to the lateral tail veins, were identified by injecting 0.01 mL of 0.1% Evans blue dye (Solarbio, Beijing, China) into the tail and subsequently ligated using 9–0 nylon sutures. Following surgery, 0.01 mL of indocyanine green (ICG; 1 mg/mL) was injected at the same site as the Evans blue dye to visualize the lymphatic drainage ( Video 1 ) and confirm successful ligation, as evidenced by fluorescence retention at the ligation site.

### Procedure for Identification and Isolation of Gluteal Lymph Nodes

**Video 2** Identification of gluteal lymph node.


**Fig. 1:**
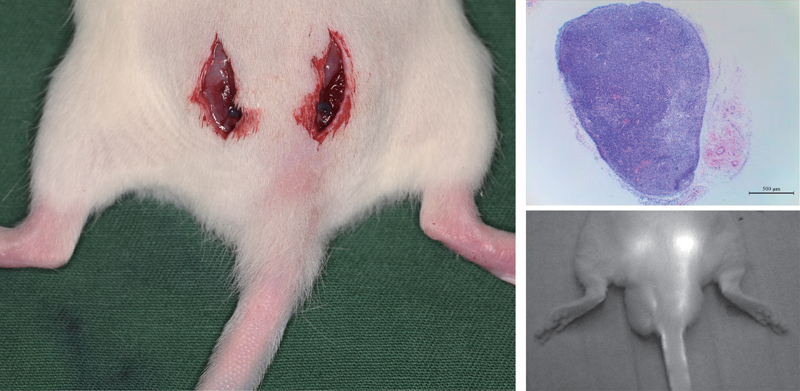
Identification and characterization of gluteal lymph nodes. The image (left) displays the bilateral gluteal lymph nodes stained with Evans blue dye, located symmetrically in the gluteal region of the subject. The image (upper-right) shows an H&E-stained tissue, confirming its identity as lymph nodes through histological examination. The image (lower-right) illustrates an indocyanine green fluorescence image, demonstrating that the gluteal lymph nodes are responsible for draining lymphatic fluid from the tail region.

To locate the GLN, 0.01 mL of ICG (1 mg/mL) was injected subcutaneously into the tail, marking the GLN locations. This was followed by a subcutaneous injection of 0.01 mL of 0.1% Evans blue dye into the tail, visualizing the GLN. The marked gluteal area was carefully incised, and the gluteus superficialis muscle was dissected to expose the clearly blue-stained GLN and surrounding lymphatic tissue, which were then excised ( Fig. 1 and Video 2 ). The gluteus superficialis muscle and skin were sutured with 6–0 nylon thread.

### Measurement of Tail Circumference and Volume

Tail diameter was measured using a Vernier caliper, starting from the distal margin of the tail and ending 6 cm from this point. Serial circumferences (C) were recorded, and tail volume was calculated weekly using the truncated cone formula: V = 14π (C _1_ C _2_ + C _2_ C _3_ + C _3_ C _4_ )V. 12

### Histological Assessment of the Tail

The harvested tails were fixed in 4% paraformaldehyde for 24 to 48 hours, then decalcified for 7 to 10 days at 37 °C using 10% EDTA. Samples ( *n* = 4) were subsequently embedded in paraffin, sectioned, and stained with Masson's trichrome.

### Bulk RNA-sequencing

Bulk RNA-sequencing was conducted on total RNA extracted from rat tail tissues (Sup group, *n* = 3; Sup + LN group, *n* = 3). A total of 1 μg RNA per sample was used for library preparation. Sequencing libraries were generated using the NEBNext® Ultra™ RNA Library Prep Kit for Illumina® (NEB, MA) according to the manufacturer's protocol, with index codes assigned to each sample for sequence attribution. mRNA was purified from total RNA using poly-T oligo-attached magnetic beads, followed by fragmentation using divalent cations and high temperature. First-strand complementary DNA (cDNA) synthesis was carried out with random hexamer primers and Moloney Murine Leukemia Virus (M-MuLV) Reverse Transcriptase. The second strand was synthesized using DNA Polymerase I and RNase H. Overhangs were converted to blunt ends, and the 3′ ends were adenylated before ligation of NEBNext adaptors with hairpin loop structures. cDNA fragments of 250 to 300 bp were selected using the AMPure XP system (Beckman Coulter, United States). Size-selected, adaptor-ligated cDNA was treated with the Uracil-Specific Excision Reagent (USER) enzyme, followed by PCR amplification with Phusion High-Fidelity DNA polymerase. PCR products were purified, and library quality was assessed using the Agilent Bioanalyzer 2100 system.

### Functional Enrichment Analysis

Functional enrichment analysis was conducted on cancer related lymphedema (CRL)-associated differentially expressed genes (DEG), with particular focus on genes expressed in Adipose-Derived Stem Cells (ADSC). Gene Ontology (GO) and Kyoto Encyclopedia of Genes and Genomes (KEGG) enrichment analyses were conducted using the clusterProfiler package, with adjusted *p* -values <0.05 considered statistically significant.

### Statistical Analysis

Data are presented as mean ± standard error. Overall differences between groups were determined using one-way analysis of variance or the Kruskal–Wallis test. Statistical significance was set at *p* < 0.05. Analyses were performed using SPSS version 29.0 (IBM, New York, NY).

## Results

### Detection of Gluteal Lymph Node

Based on the ICG imaging, the GLNs are located bilaterally in the gluteal region and are connected to the LVs of the tail, serving as the drainage pathway for the tail. This observation aligns with previously identified lymphosome territories of the tail. Following an injection of Evans blue dye into the tail, dissection of the gluteal region revealed blue-stained tissues. Subsequent H&E staining of these tissues confirmed the distinct structure of GLN ( Fig. 1 ).

### Circumference and Volume of Tail Model

**Fig. 2:**
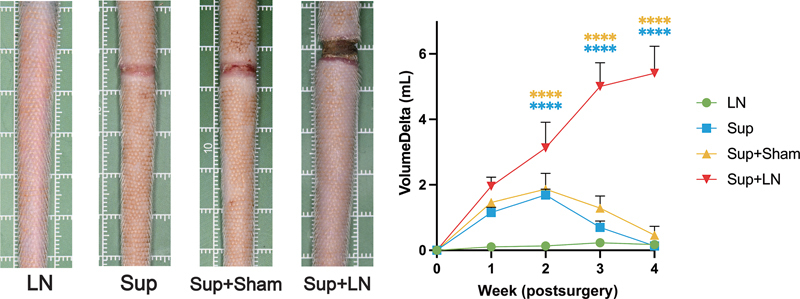
Tail volume changes and representative images postsurgery. The graph (left) shows tail volume changes over four weeks in LN, Sup, Sup + Sham, and Sup + LN groups. (Right) The Sup + LN group exhibited the most significant and sustained volume increase, indicating severe lymphedema development. In contrast, the Sup and Sup + Sham groups showed moderate increases, stabilizing after week 2. The LN group remained stable with minimal changes. * *p* < 0.05; ** *p* < 0.01; *** *p* < 0.001; **** *p* < 0.0001.

The tail volume changes of the Sup, Sup + LN, Sup + Sham, and LN groups exhibited significant differences after the operation (within 4 weeks). The Sup + LN group exhibits the most significant volume increase, which continued to increase throughout the 4-week observation period, indicating that tail skin removal combined with GLN removal promotes sustained lymphedema development. In contrast, the Sup, Sup + Sham, and Sup + LN groups show moderate volume increases, and their volume changes stabilize after week 2. Starting from week 2, the volume increase in the Sup + LN group became statistically significantly different from that of the Sup and Sup + Sham groups, further highlighting the impact of combined lymph node and skin removal on lymphedema progression. The LN group remains relatively stable across the postoperative weeks, with minimal volume changes, indicating that GLN removal alone does not significantly contribute to volume increase without the added factor of tail skin or deep lymphatic disruption ( Fig. 2 ).

**Fig. 3:**
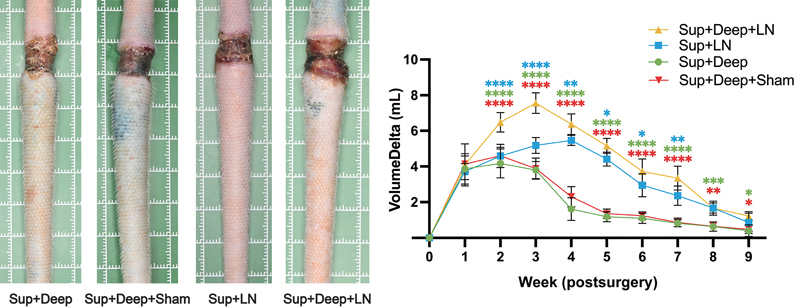
Postoperative tail volume difference. The graph shows postoperative tail volume changes in Sup + Deep + LN, Sup + Deep, Sup + Deep + Sham, and Sup + LN groups over 6 weeks. Representative tail images (left) at week 3 highlight the differences in swelling severity across groups. (Right) The Sup + Deep + LN group displayed the most significant swelling, peaking at week 3, followed by a gradual decrease, but with no significant long-term difference compared to the Sup + LN group. The Sup + Deep and Sup + Deep + Sham groups showed moderate, transient swelling peaking at week 2 and resolving thereafter. * *p* < 0.05; ** *p* < 0.01; *** *p* < 0.001; **** *p* < 0.0001.

The postoperative tail volume data reveal distinct patterns of lymphedema progression among the groups (Sup + Deep + LN, Sup + Deep, Sup + Deep + Sham, and Sup + LN), highlighting the impact of different interventions on swelling dynamics. The Sup + Deep group, involving both superficial tissue removal and deep lymphatic ligation without lymph node excision, displays a moderate swelling response that peaks early at week 2 before gradually declining. Similarly, the Sup + Deep + Sham group, which is a sham control with preserved GLN, follows a comparable trajectory to the Sup + Deep group. Both groups experience moderate and transient volume increases, peaking around week 2 and then steadily decreasing. These findings suggest that deep lymphatic ligation alone primarily contributes to short-term edema but lacks the capacity to sustain prolonged lymphedema. In contrast, the Sup + Deep + LN group, which combines superficial tissue removal, deep lymphatic ligation, and GLN excision, exhibits the most pronounced swelling response, reaching a substantial peak at week 3. While a gradual reduction in volume is observed after this peak, the difference in volume between the Sup + Deep + LN and Sup + LN groups diminishes in the later weeks, ultimately showing no statistically significant variation in week 8. This convergence suggests that, although the addition of deep lymphatic ligation in the Sup + Deep + LN group initially intensifies the edema response, its long-term impact fades, leading to similar sustained swelling levels as seen in the Sup + LN group, where only superficial tissue removal and lymph node excision were performed ( Fig. 3 ).

### Fibroadipose Thickness and Fibrosis of Subcutaneous Tissue in the Tail Model

**Fig. 4:**
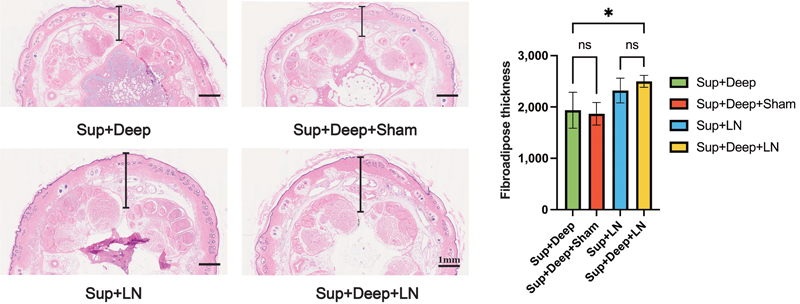
Postoperative tail fibroadipose thickness. H&E staining of tail sections collected at week 6 reveals significant fibroadipose thickening in the Sup + Deep + LN group compared to the Sup + Deep group. The Sup + LN group also displayed fibroadipose thickening, with no statistically significant difference compared to the Sup + Deep + LN group. * *p* < 0.05; ** *p* < 0.01; *** *p* < 0.001; **** *p* < 0.0001.

H&E staining of samples collected at week 6 revealed significant fibroadipose thickening in the Sup + Deep + LN group compared to the Sup + Deep group ( *p* < 0.05). While the Sup + LN group also showed fibroadipose thickening, there was no statistically significant difference between the Sup + LN and Sup + Deep + LN groups. Representative images confirm that the Sup + Deep + LN group exhibited the thickest fibroadipose layer overall ( Fig. 4 ). Masson's staining of week 6 samples demonstrated increased collagen deposition in the Sup + LN and Sup + Deep + LN groups, with the Sup + Deep + LN group showing the most pronounced fibrosis ( Supplementary Fig. S3 [available in the online version only]).

### Pathophysiology Changes after Gluteal Lymph Node Removal

**Fig. 5:**
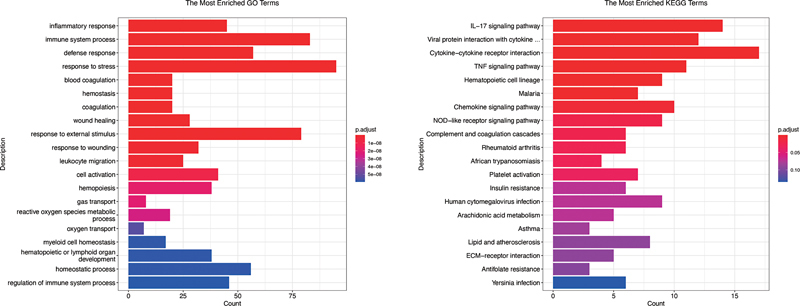
Gene Ontology (GO) and Kyoto Encyclopedia of Genes and Genomes (KEGG) enrichment analyses of differentially expressed genes between Sup + LN and Sup groups following gluteal lymph node removal. The top enriched GO biological process terms indicate significant activation of immune-related processes, including inflammatory response, immune system activation, leukocyte migration, and cytokine-mediated signaling, as well as pathways involved in wound healing and fibrotic remodeling (left). The most enriched KEGG pathways show upregulation of IL-17, TNF, and cytokine–cytokine receptor interaction pathways, as well as hematopoietic and chemokine signaling pathways, all contributing to immune dysregulation and fibrosis (right).

The GO and KEGG enrichment analyses comparing the Sup + LN and Sup groups revealed several significant GO and KEGG terms, including inflammatory cytokine (IL-17 and TNF) and immune infiltration. Besides, adipose tissue metabolic process and extracellular matrix organization were enriched in GO and KEGG analysis ( Fig. 5 ).

### Postoperative Indocyanine Green Lymphography of Integrated Rat Tail Model

Six weeks after the integrated surgical intervention, which included circumferential superficial tissue excision, deep lymphatic ligation, and GLN dissection, ICG lymphography revealed significant changes in lymphatic flow dynamics ( Supplementary Fig. S4 [available in the online version only]). In the region of circumferential excision, ICG fluorescence was unable to progress proximally along the superficial LVs, instead stagnating at the excision site, indicating complete disruption of superficial lymphatic flow. However, the deep lymphatic system exhibited partial recanalization, as evidenced by the proximal flow of ICG fluorescence, suggesting compensatory drainage through the deep LVs.

## Discussion

In this study, we successfully established a novel and reproducible rat tail lymphedema model by integrating circumferential superficial tissue excision, deep lymphatic ligation, and GLN removal. Human secondary lymphedema is closely linked to cancer-related lymphadenectomy, where lymph nodes play dual roles as critical hubs for lymphatic drainage and immune regulation. These roles are evident in clinical settings, where vascularized lymph node transfer is utilized to restore disrupted lymphatic pathways, improve lymphatic drainage, reduce limb swelling, and lower infection recurrence rates. 13 14 However, previous rat tail models failed to incorporate lymph node removal, limiting their ability to mimic the pathophysiology of human secondary lymphedema fully. To address this gap, we developed a lymphosome-based rat tail model, identifying the GLNs as the primary drainage pathway for tail lymphatics using ICG imaging and histological analysis. This model allowed us to design targeted interventions to explore the connections between lymphatic systems and assess their contributions to lymphedema severity and progression.

In previous rat tail lymphedema models, edema is commonly induced by circumferential skin excision and/or deep LV ligation. These models typically exhibit peak swelling between postoperative day 1 and week 3, with some reporting edema lasting up to 4 weeks when only skin excision is performed. 10 11 15 16 17 18 However, the absence of lymph node removal in these models often results in transient and reversible edema, making them inadequate for simulating the chronic pathophysiology of secondary lymphedema observed in patients after oncologic lymphadenectomy. In our study, the Sup + Deep and Sup + Deep + Sham groups followed a similar short-term edema trajectory, suggesting that isolated disruption of superficial and deep lymphatics is insufficient to sustain long-term swelling, likely due to the robust regenerative and compensatory capacity of the rodent lymphatic system. To address these limitations, we developed a composite, lymphosome-guided model incorporating three key interventions: GLN excision, deep lymphatic ligation, and circumferential skin excision. This combinatorial strategy successfully established a more persistent and clinically relevant model of chronic lymphedema. Notably, groups involving GLN removal exhibited more pronounced and longer-lasting edema, underscoring the essential role of lymph node dissection in sustaining chronic lymphedema and closely mirroring the disease progression observed following cancer-related lymphadenectomy in clinical settings. The value of our model is further supported by findings from previous systematic reviews, 19 20 21 highlighting that most rodent models without lymph node intervention induce only short-lived edema, typically resolving within 1 to 3 weeks due to the animals' high lymphatic regenerative capacity. While large animal models more accurately reflect human lymphatic anatomy and pathology, they are often limited by ethical concerns and financial cost. Our approach retains the advantages of small-animal experimentation—such as scalability and cost-effectiveness—while significantly improving the ability to replicate the chronic features of human lymphedema.

Our findings revealed that lymph node excision is pivotal for sustaining lymphedema. Groups with lymph node removal ( Fig. 2 ) demonstrated prolonged and severe swelling compared to groups without lymph node involvement. However, lymph node removal alone was insufficient to induce edema. The inability of lymph node removal alone to induce chronic lymphedema may be related to the lymphangiogenesis process that follows nodal disruption, compensating to preserve drainage. 15 ICG lymphography revealed lymphangiogenesis in the gluteal region where lymph nodes were removed, highlighting the formation of new lymphatic networks connecting the tail lymphatics to the popliteal lymph nodes, releasing the edema ( Supplementary Fig. S4 [available in the online version only]). Therefore, the superficial lymphatic drainage system of the rat tail is disrupted following circumferential skin excision. However, the presence of the deep lymphatic system compensates for the loss of superficial lymphatic circulation, preventing the development of lymphedema. As a result, skin excision alone does not induce significant lymphatic dysfunction but rather creates a transient wound on the rat tail. The removal of GLN triggers a sequence of pathophysiological processes, such as inflammation, fat deposition, and fibrosis, which closely to human lymphedema 22 23 ( Fig. 5 ). In contrast, deep lymphatic ligation has been widely demonstrated in previous studies to result in lymphedema; however, in cases involving deep lymphatic ligation, the gradual reduction within in tail volume within 4 weeks, may related to the formation of compensatory lymphatic bridges or collateral pathways. These pathways partially restored lymphatic drainage, narrowing the differences between the Sup + Deep + LN and Sup + LN groups in later weeks.

This regenerative capacity necessitates the simultaneous disruption of both lymph nodes, superficial lymphatics, and deep lymphatics for sustaining swelling. In summary, our study identified a stepwise progression in lymphedema severity with each additional intervention. (1) Superficial skin tissue removal: This disrupted initial lymphatic flow, resulting in transient edema that resolved within 2 weeks. This rapid resolution may from the robust regenerative or compensatory capacity of the lymphatic system. (2) Deep lymphatic ligation: Deep lymphatic disruption contributed to acute, transient swelling, characterized by an early peak but an inability to sustain long-term lymphedema without accompanying lymph node removal. (3) Lymph node removal: Lymph node excision was critical for maintaining prolonged edema. Furthermore, the disruption of normal lymphatic drainage pathways following GLN dissection led to profound alterations in lymphatic architecture and function. Under ICG lymphography, the LVs of the tail exhibited a characteristic “string-of-beads” appearance, reflecting impaired lymphatic flow, segmental dilation, and potential lymphatic stasis ( Supplementary Fig. S4 [available in the online version only]). The Sup + Deep + LN group, which combined all three interventions, exhibited the most severe and sustained lymphedema.

Otherwise, histological analyses and bulk RNA-sequencing further substantiated the progression to fibrosis, which mirrors the chronic phases of human lymphedema. In Sup + LN, Sup + Deep, and Sup + Deep + LN groups, dense collagen deposition and fibrotic changes were observed in subcutaneous tissues, reflecting the development of subcutaneous fibrosis, indicating the essential role of simultaneous lymphatic disruption and node removal and deep lymphatic ligation in driving fibrotic progression ( Supplementary Fig. S3 [available in the online version only]). These observations validate the model's ability to simulate lymphedema pathology, including fibrotic remodeling.

Our novel model, validated through both histological analysis and ICG lymphography, provides a comprehensive platform for studying the mechanisms underlying lymphedema. Observations of lymphatic disruption, recanalization, and lymphangiogenesis further enhance its utility in evaluating potential therapeutic interventions such as VLNT, VLVT, decellularized scaffolds, and biomaterials. 24 25 By bridging the gap between experimental findings and clinical applications, this model addresses the limitations of previous approaches and supports the development of tailored strategies for different lymphedema presentations. Although we did not perform a formal power calculation, our group sizes were determined based on prior studies and established practices in rodent lymphedema models. The sample size was carefully chosen to balance ethical principles with statistical feasibility. We recognize that the moderate number of animals per group may pose limitations in detecting subtle differences and suggest that future studies could benefit from larger-scale validation. Moreover, the layered effects of lymphatic disruption demonstrated in our study suggest opportunities for refining models to simulate varying severities and durations of lymphedema based on clinical needs.

## Conclusion

This study presents a novel lymphosome-based rat tail lymphedema model that incorporates lymph node removal. The findings emphasize the critical role of lymph node involvement in maintaining long-term edema, with deep lymphatic ligation amplifying early swelling. This model offers valuable insights into the mechanisms and progression of lymphedema and may serve as a model for evaluating targeted interventions and therapeutic strategies.
