# Supplementary material for: A Novel Lymphosome-Based Long-Lasting Rat Tail Model of Lymphedema
Source: J Reconstr Microsurg. 2025 Sep 3;42(6):466–72. doi: 10.1055/a-2687-0506 (PMC13286090; doi:10.1055/a-2687-0506)
Supplement: Supplementary file 3 — Supplementary Material [file 10-1055-a-2687-0506-s25050114.pdf]

A novel lymphosome-based tail model

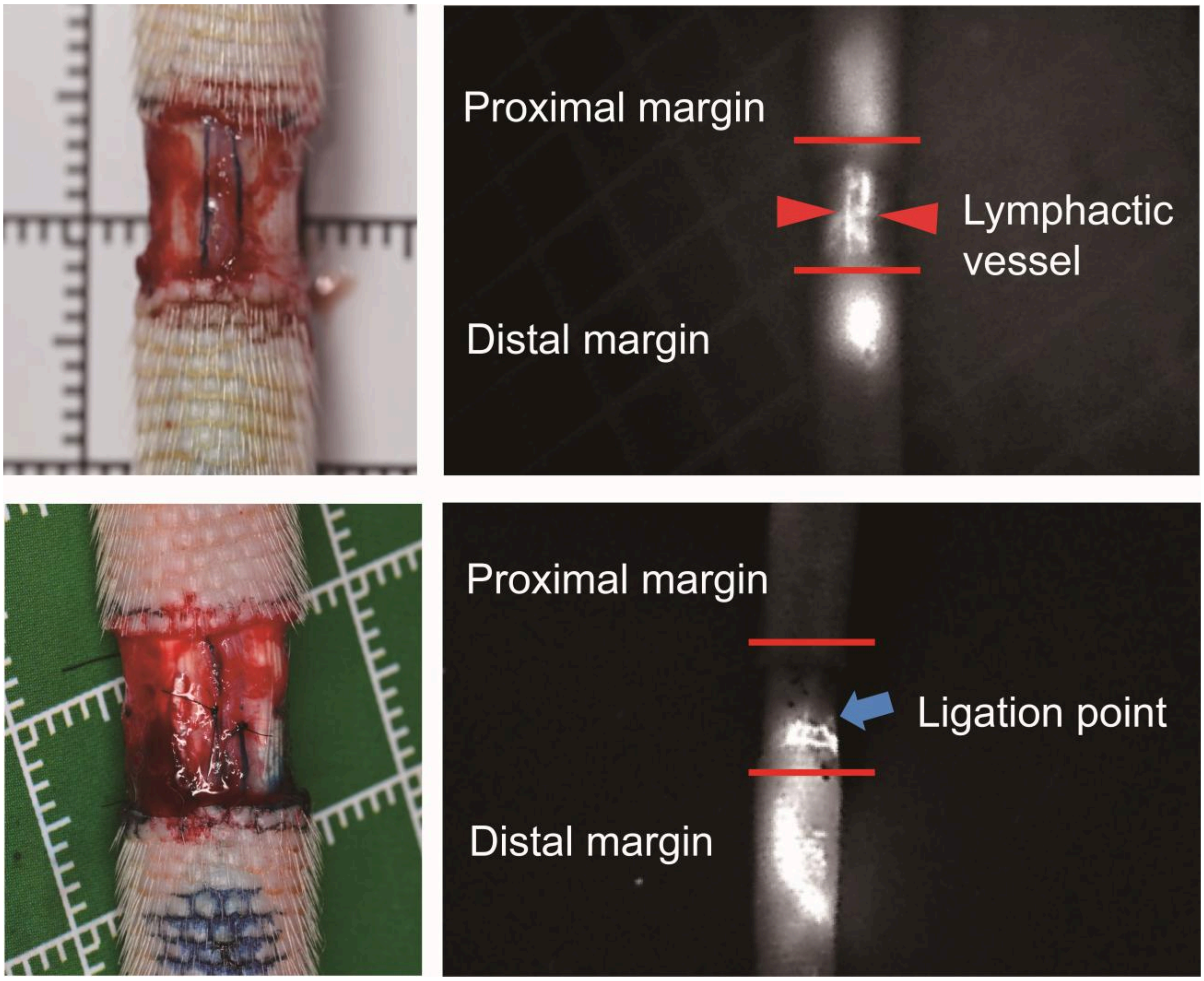

Supplementary Fig. S1 Anatomical and functional imaging of lymphatic vessels in the mouse tail. The pictures(top) illustrate the anatomy lymphatic vessels in the mouse tail. The image (top left) shows the exposed lymphatic vessels stained with Evans Blue dye. The image (top right) shows an ICG (indocyanine green) fluorescence image, highlighting the lymphatic vessel (red arrows). The flow of lymphatic fluid from the distal to the proximal margin is evident under normal conditions. The bottom pictures depict the lymphatic vessel post-ligation. The image (bottom left)

Journal of Reconstructive Microsurgery | DOI 10.1055/a-2687-0506 | © The Author(s).

shows the site of ligation of the lymphatic vessel. The corresponding ICG fluorescence image （bottom right） confirms that the ligation successfully obstructed lymphatic flow (blue arrow), as lymphatic fluid is no longer able to move from the distal margin to the proximal margin.

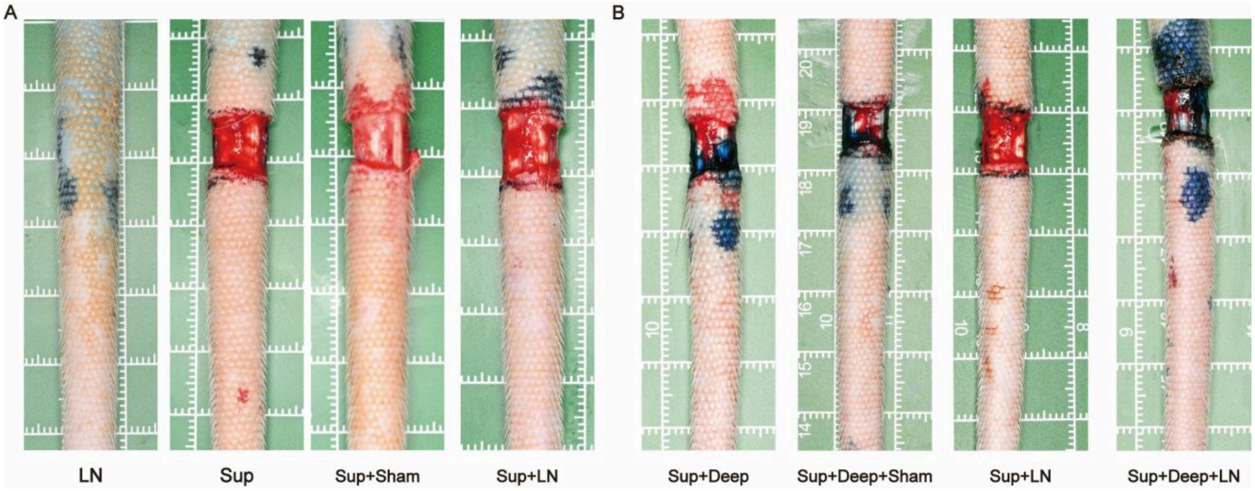

Supplementary Fig. 2 Immediate postoperative images of rat tails across experimental groups. (A) Immediate postoperative images of groups: LN, Sup, Sup+Sham, and Sup+LN. (B) Immediate postoperative images of groups: Sup+Deep, Sup+Deep+Sham, Sup+LN, and Sup+Deep+LN.

A novel lymphosome-based tail model

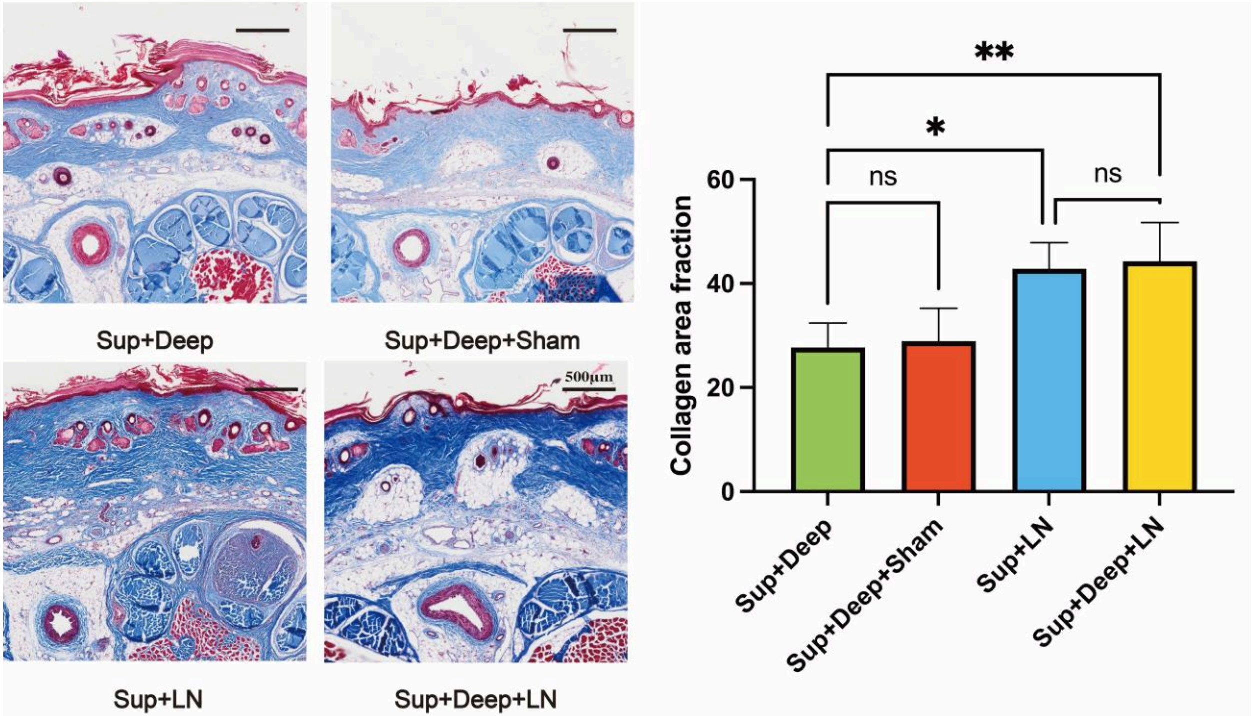

Supplementary Fig. 3 Postoperative tail fibrosis condition. Masson's trichrome staining of week 6 samples demonstrates increased collagen deposition in the Sup+LN and Sup+Deep+LN groups, with the Sup+Deep+LN group showing the most pronounced fibrosis.

Journal of Reconstructive Microsurgery | DOI 10.1055/a-2687-0506 | © The Author(s).

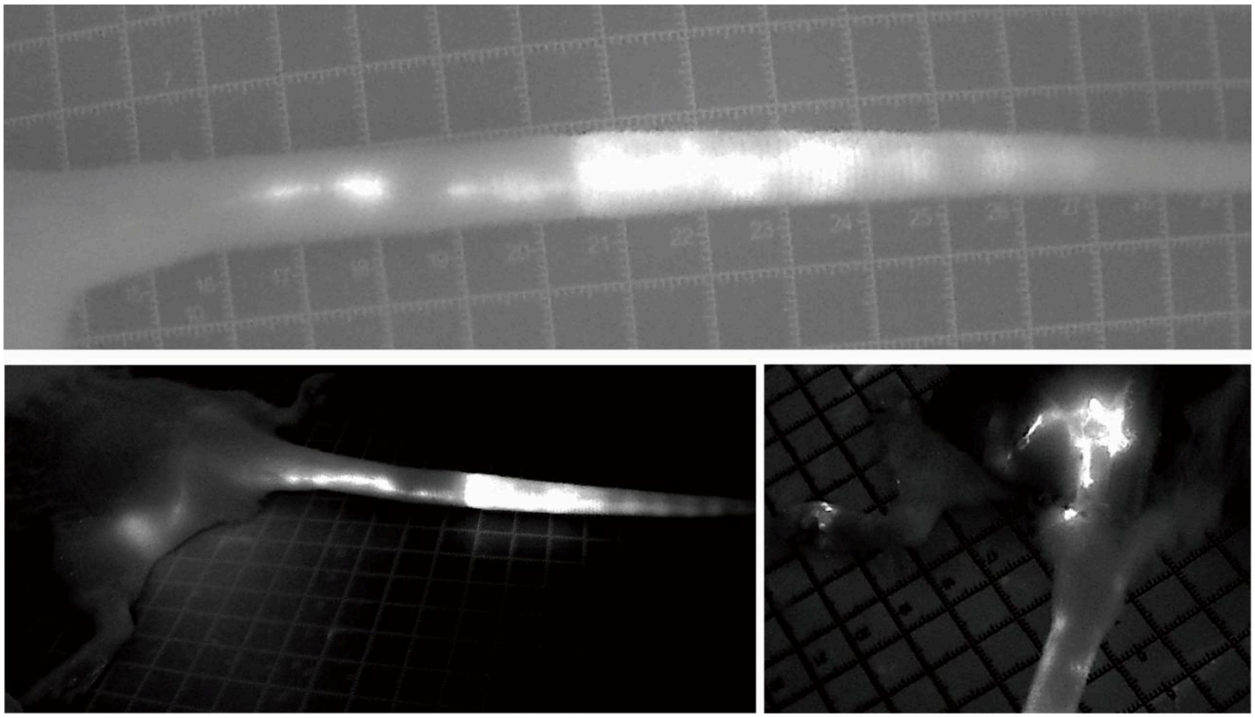

Supplementary Fig. 4 Postoperative lymphatic flow dynamics observed using ICG lymphography. Six weeks after the combined surgical intervention—including circumferential superficial tissue excision, deep lymphatic ligation, and GLN dissection—ICG lymphography revealed significant changes in lymphatic flow(upper). In the region of circumferential excision (bottom left), ICG fluorescence was unable to advance proximally along the superficial lymphatic vessels, instead stagnating at the excision site, indicating complete disruption of superficial lymphatic flow. In contrast, the deep lymphatic system showed partial recanalization, and lymphatic network was reestablished in GLN removed area(right).

Journal of Reconstructive Microsurgery | DOI 10.1055/a-2687-0506 | © The Author(s).
